# The Future Is Open: Open-Source Tools for Behavioral Neuroscience Research

**DOI:** 10.1523/ENEURO.0223-19.2019

**Published:** 2019-08-09

**Authors:** Samantha R. White, Linda M. Amarante, Alexxai V. Kravitz, Mark Laubach

**Affiliations:** 1 Center for Behavioral Neuroscience, American University, Washington, DC 20016; 2 Department of Psychiatry, Washington University, St. Louis, MO 63130

**Keywords:** behavior, designs, methods, open source, protocols, tools

## Significance Statement

There has been a recent and substantial increase in the use of open-source tools for conducting research studies in neuroscience. The OpenBehavior Project was created to disseminate open-source projects specific to the study of behavior. In this commentary, we emphasize the benefits of adopting an open-source mindset and highlight current methods and projects that give promise for open-source tools to drive advancement of behavioral measurement and ultimately understanding the neural basis of behavior.

## 

Over the past decade, there has been an explosion in the use of new neurobiological tools for measuring and controlling brain cell activity. Recent developments in optogenetics, chemogenetics, cellular imaging, and fiber photometry have spiked publications across cellular, systems, and behavioral neuroscience. Researchers with expertise in molecular biology or cellular physiology are now carrying out behavioral studies, and often bring a fresh approach to the fine-grained study of behavior that has led to the development of many new assays for measuring behavior and cognition in animal models (mice, flies, worms, etc.).

Thanks to a revolution in low-cost methods for 3D printing and off-the-shelf microcontrollers (e.g., Arduino, Teensy, microPython) and single-board computers (Raspberry Pi), many of these research groups are able to create complex behavioral tasks quite easily. The R and Python languages, specialized computing libraries (e.g., numpy, OpenCV, TensorFlow), and the Anaconda Python distribution have been crucial for the development of open source analysis software for neuroscience projects. In parallel, these developments in neuroscience research have occurred during a time when there is a simultaneous movement toward sharing computer code (Eglen et al., 2017; Gleeson et al., 2017), through websites like GitHub, and opening up the process of software and hardware design to non-experts through hackerspaces and makerspaces.

Despite these developments, there is still room for growth with regards to sharing. Designs for some new tools have been posted on websites created by individual researchers or shared via public repositories such as GitHub. In other cases, designs and protocols have been published and several new journals and tracks in existing journals are emerging for reports on open source hardware and software. In this commentary, we aim to emphasize the benefits of adopting an open source mindset for the behavioral neuroscience field, and we highlight current methods and projects that give promise for open source tools to drive advancement of behavioral measurement and ultimately understanding the neural basis of these behaviors.

## Why Open Source?

The main idea behind an open source project is that the creator or developer provides open access to the source code and design files, whether that be for software or hardware. Open source projects typically provide a license for others to use and modify the design, although many licenses require that any modifications remain open source. Under such licenses, it is not permissible to take an open source design, modify a few things, and claim it is a new closed design. Releasing a project with an open source license provides transparency for others to view, modify, and improve the project. Open source can be relevant for many levels of scientific research; open-access journals, code and data repositories, and sharing methods, protocols, or files are all examples of how one can contribute to open source science.

**Figure 1.**
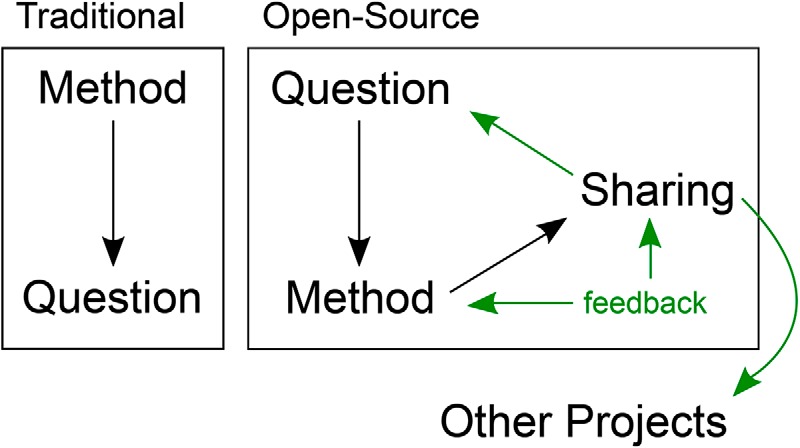
Open source creative process: methods and questions. Traditional methods in neuroscience are purchased commercially and are used to answer a specific research question. Due to the need to maximize use based on the cost of the tool, the method often drives subsequent research questions. However, in an open source model, the research question drives the development of a method or tool. A major advantage of this in behavioral neuroscience is that previously unmeasured aspects of behavior now have the potential to be measured, leading to a new frontier of behavioral measurement and analysis. The tool is subsequently shared to the community, and the user seeks feedback from the community to refine the method. Sharing of an open source tool leads to the development of new projects across multiple research labs, leading researchers to, quite literally, think “outside the box.”

The term “open source” is also often synonymous with being cost-effective. Many commercial products used in neuroscience can be replicated in an open source manner at a fraction of the initial cost. However, there are additional advantages to incorporating open source science in a research lab. With a recent increase in microcontrollers, microprocessors, 3D printing and laser-cutting technologies, most people now have access to create devices or products in a way that was previously unavailable to researchers. Additionally, a major benefit to open source science is the ability for customization and flexibility. Instead of being restricted to studying only what a commercial part is capable of doing or measuring, it is now possible to study a level deeper through developing a device or software that will help answer the research question, instead of letting the technology drive the research question (Fig. 1). In behavioral neuroscience, this allows researchers to enter uncharted territory of analyzing previously unmeasured or fine-grained aspects of behavior (Krakauer et al., 2017).

Several extremely successful projects have come from this open source movement (Maia Chagas, 2018), including neuroscience projects such as the Open Electrophysiology project (Siegle et al., 2017), the UCLA miniaturized microscope (Aharoni et al., 2019), and software such as Bonsai (Lopes et al., 2015) and DeepLabCut (Mathis et al., 2018) for video recordings and analysis. However, the field of open source neuroscience is expanding at a rapid pace, and it is becoming hard to keep up with all the latest advances in research tools and the hardware and software that has enabled them.

## The OpenBehavior Project

In 2016, it became clear that there were many projects reporting on new tools for the study of behavior, and thus we launched the OpenBehavior project. Access to design files and build instructions relied on word of mouth and isolated blogs and posts on social media. We made it our goal to disseminate information about tools as soon as they emerge as preprints on bioRxiv or PsyArXiv, peer-reviewed manuscripts, or independent posts by developers on Hackaday, GitHub, lab websites, or social media. The project is based around a website covering bleeding-edge open source tools and a related Twitter account that keeps followers up-to-date with new projects relevant to behavioral neuroscience in species from flies and fish, to rodent and, more recently, humans. Through these efforts, we hope to contribute to the rapid replication and adoption of new tools into ongoing research and trigger modifications of existing tools for novel research applications.

**Figure 2.**
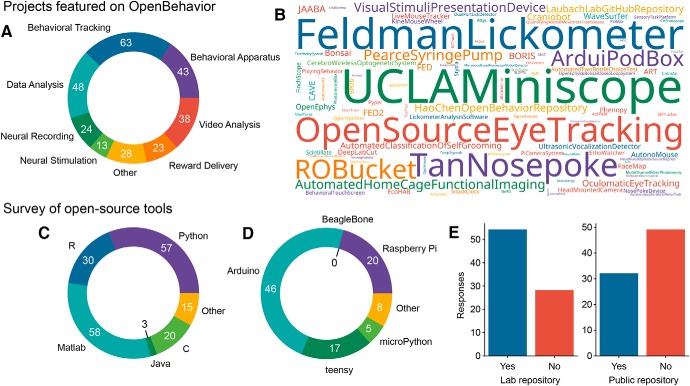
Projects featured on OpenBehavior and a survey of our followers. ***A***, Types of projects featured on OpenBehavior. The most common type of project allows for tracking behaviors in video recordings. Most projects have multiple functions. For example, Bonsai can be used for video recording, tracking behaviors, and controlling behavioral equipment. ***B***, Based on web hits from unique URLs, we depict the overall interest of our followers. ***C***, A survey on use of open source tools revealed that most labs use more than one programming language, with MATLAB/Octave and Python most commonly used. ***D***, The survey also found that the majority of respondents reported using Arduinos microcontrollers, and less common tools included Raspberry Pi single board computers and Teensy microcontrollers. ***E***, The majority of respondents reported having repositories for code and designs in their labs. However, most of these researchers did not report use of public repositories.

To date, dozens of projects have been shared through www.openbehavior.com, with even more shared through active Twitter engagement. In May 2019, we celebrated our 100th open source project post, which have covered devices for delivering rewarding foods and fluids, measuring home cage activity, video tracking and analysis, and physiologic methods used in behavioral experiments such as miniaturized microscopes and fiber photometry (Fig. 2*A*,*B*). While video analysis is a prominent focus of many projects, several other types of projects have been popular on the site, including devices for tracking patterns of feeding behavior in the home cage (FED; Nguyen et al., 2016), a system for multi-channel electrophysiology (OpenEphys; Siegle et al., 2017), systems for fiber photometry (PhotometryBox; Owen and Kreitzer, 2019), stimulators for optogenetic stimulation (Stimduino; Sheinin et al., 2015), supervised (JAABA; Kabra et al., 2013; DeepLabCut; Mathis et al., 2018), and unsupervised (FaceMap; Stringer et al., 2019) machine-learning algorithms for analyzing behaviors from video, and integrated systems for behavioral control (Bpod; RRID:SCR_015943) including video recording and real-time analysis (Bonsai; Lopes et al., 2015). Recently, we have begun to track and share tools for research in human behavioral neuroscience, computational models, and relevant data analysis methods.

## Sharing and Dissemination of Open Source Tools

Thanks to the sharing of proper documentation and an understanding of open source methods, researchers were able to modify some of the projects to better fit their experiments’ needs. One example of how open source tools can lead to new research projects is found in some of the earliest posts on OpenBehavior. We featured a number of devices for delivering rewarding fluids to rodents. One project, the Automated Mouse Homecage Two-Bottle Choice Test by Dr. Meaghan Creed, was developed to allow for automated taste preference tests and oral self-administration studies in mice. The project was posted to a website for sharing the designs of open source hardware (Hackaday.io) and the device was quickly used by a number of labs. One of these labs, with knowledge of open source methods and insightful documentation from Creed, was able to modify the device using a more advanced microcontroller which allowed them to measure fluid consumption over 16 reward tubes simultaneously in rats (Frie and Khokhar, 2019). The experiences of these users of our website and followers of our Twitter feed indicate that we have had strong initial success in our overall mission to accelerate research through promotion of collaboration and sharing.

To assess how OpenBehavior might further improve sharing and dissemination, in the spring of 2019, we conducted an online survey. While not a scientific poll, the results are informative about the views and needs of the open source community of behavioral neuroscientists; 50% of respondents (48 out of 70) indicated that they follow the site with the intention to incorporate some of the devices and software that we have profiled into their research programs in the future. Another 30% of survey respondents (22 out of 70) indicated they have used tools featured on the site that were not developed by their own labs either straight from the project documentation (16 out of 22) or with some modifications of their own (six out of 22). Many participants who reported integration of open source tools into their research programs have often incorporated more than one, which has generated their own documented method for recording and analyzing behavior (van den Boom et al., 2017) or generated full closed-loop systems for behavioral experiments (Buccino et al., 2018; Solari et al., 2018).

Further efforts on dissemination and training are needed to further the impact of OpenBehavior and related projects within the research community. We are exploring adding a forum to the website to encourage interactions between developers and users, which was suggested by several participants of our survey. Furthermore, we would like to inspire DIY hackers and open source engineers to think about projects that could be useful for behavioral neuroscience, just as we’ve begun to seek hackers to make sense of large datasets in neuroscience (Goodwin, 2015). To this end, we have initiated efforts through a partnership with Hackaday.io, a website that is popular in the DIY community.

## Expanding Adoption of Open Source Tools

Despite these advantages of open source tools, incentives to sharing and the ability to categorize and disseminate developments remains a challenge. Worse, there are major technical barriers that hold many researchers back from diving headfirst into a newly released research tool. Not everyone has the incentive, skills, or time needed to incorporate new tools into ongoing research projects. It takes time to learn the skills required to build new devices and programs from source. Clear instructions from developers are further needed to recreate and use new devices and programs. Concerns persist about the reliability of self-made devices or undiscovered bugs in programs written for relatively small user bases. The lack of immediately available technical support and extensive validation of new tools does not add positively to confidence in using new open source tools.

Notwithstanding these concerns, there has been movement toward to the use of open source software and hardware in neuroscience as well as evidence for sharing new tools by neuroscience labs. To assess how followers of OpenBehavior make use of software and hardware in their research, we ran a second on-line survey in late May 2019 that queried respondents about the programming languages used in their labs, their use of microcontrollers, 3D printers, and printed circuit boards, and also whether they used in-lab and/or public repositories for their code and designs. Findings from the survey are described in Figure 2*C–E*. Remarkably with regards to sharing, while most (65%) respondents reported having repositories for their labs (54 of 82), <40% of respondents (32 of 81) reported sharing their code and designs on public repositories.

These findings are relevant in the light of ongoing discussions about the availability of neural data and analysis code (Halchenko and Hanke, 2012; Ascoli et al., 2017; Eglen et al., 2017; Gleeson et al., 2017), and open sharing of new methods for data collection [OpenEphys (Siegle et al., 2017); UCLA miniscope (Aharoni et al., 2019)]. We hope that this will lead to new conversations about sharing behavioral data, analysis code, and hardware. It seems straightforward to encourage an open source mindset, which can be done across several levels. Anyone should be able to replicate an open source project, given they are provided with detailed documentation and dissemination of software or hardware devices. It is necessary to encourage a set of standards to make reproducibility possible, such as in the methods for two-bottle preference testing described above. See Box 1 for our recommendations for best practices in developing open source tools.

Box 1. **Recommendations for best practices in developing open source tools**

**1. Clear documentation of the project.** Provide all design files, as well as a bill of materials, build instructions, graphical (video/photo/3D renderings) descriptions or tutorials for the project.

**2. Central repository for files.** Provide all files and documentation of the project on a site like GitHub, Hackaday.io, OSF.io, or on the research group’s website.

**3. Experimental validation.** Show an example of the device being used in a behavioral experiment.

**4. Make the project easy to find.** Create a Research Resource Identifier (RRID), using the SciCrunch project, for the device so that others can track the project across publications.

Additional efforts are needed to offer and maintain productivity using open source tools. There is a need for forums for public discussions on the tools, perhaps through the Neuronline forums managed by SfN. There will always be some troubleshooting, which is why an open forum for sharing feedback on already developed tools is necessary. To further drive innovation and development, we suggest implementing webinars, online tutorials, and workshops to allow people all over the world to have access to the development of open source tools. Hands-on workshops have been successful for several open source tools, such as optogenetics, CLARITY, Miniscope, and DeepLabCut. These activities will require financial support to enable storing data, designs, and protocols, maintaining a well-documented website and source code, and offering training workshops. We hope that major funders (e.g., NIH, NSF, EU) consider providing special opportunities for supporting development and training for open source research tools.

Finally, there is a need for tracking the use of open source tools, by creating and using RRIDs (SciCrunch) in publications. To our knowledge, RRIDs have not been commonly created for hardware. Having a system for tracking usage has three potential impacts. First, tool usage can be tracked beyond citations of methods papers. Second, revisions and spin-offs can be noted and also tracked. Third, developers might have increased incentives to share designs early in the process, especially if an index, similar to the h factor, was developed for RRIDs Inevitably, creating new platforms and incentives for sharing the development, use, and replication of open source behavioral tools is crucial for bringing open source science to the forefront.

## Synthesis

Reviewing Editor: Lorna Role, NINDS Decisions are customarily a result of the Reviewing Editor and the peer reviewers coming together and discussing their recommendations until a consensus is reached. When revisions are invited, a fact-based synthesis statement explaining their decision and outlining what is needed to prepare a revision will be listed below. The following reviewer(s) agreed to reveal their identity: Niraj Desai, Denise Cai.

Both reviewers found this to be an easy to read and timely commentary about the open-source movement in neuroscience. It gives a broad introduction to different open-source projects and highlights one platform, Open Behavior, which has been successful in curating and highlighting different types of open-source tools for neuroscience.

While most of the commentary is focused on the pros of open-science, it also briefly touches on the challenge of using open-source tools (for example, these tools may require more troubleshooting or require more training and are often not plug and play).

The reviewers raised a few additional queries for the authors to address and suggestions for text that might be helpful to rounding out the commentary if there is space for these considerations.

1. The authors could also include that one way to overcome some of these challenges are through hands-on workshops. Some examples that seem to have been particularly successful are Optogenetic/CLARITY workshops, Miniscope workshops, DeepLabCut workshops- all demystifying open -source tools.

2. Another challenge that the authors may consider highlighting is that open-source development and training (such as storing data for others to download, maintaining a well-documented website and source code, as well as workshops) are expensive in both time and resources consumed to do a good job. The current reality of the limited funding opportunities to support these efforts should be noted with perhaps some guidance on possible funding sources/

3. Can the authors provide more insight into how common is the use of open-source tools? The number of open-source projects seems enormous, but how many of these projects actually get used?

The online survey results give a kind of answer, but that's a self-selected group (people who visit openbehavior.com and are moved to answer an online survey are probably more into open source than other scientists are). One reason, I think, that people who could contribute to open source do not do so is the fear that nobody will ever use their tool, and so it'll be a waste of time. Are there any metrics on the use of open-source tools the authors could cite or describe?

4. What do the authors suggest as specific approaches to establishing best practices (e.g. standards and documentation) in open-source tools? In neurophysiology, there have been efforts to establish standards for data sharing (i.e., Neurodata Without Borders). For the open source project, establishing standards (even for documentation alone) would be much more difficult, given how varied projects are, but do the authors have any ideas about how this might be done? They mention the importance of documentation/standards in the manuscript, but do they have suggestions?

Minor comments:

P. 3, I think the word should be “tracks” and not “tracts”.

P.4, Teensy is normally capitalized.

## Author Response

Synthesis of Reviews: Significance Statement Comments for Author (Required): NA Comments on the Visual Abstract for Author (Required): excellent Synthesis Statement for Author (Required): Both reviewers found this to be an easy to read and timely commentary about the open-source movement in neuroscience. It gives a broad introduction to different open-source projects and highlights one platform, Open Behavior, which has been successful in curating and highlighting different types of open-source tools for neuroscience.

While most of the commentary is focused on the pros of open-science, it also briefly touches on the challenge of using open-source tools (for example, these tools may require more troubleshooting or require more training and are often not plug and play). The reviewers raised a few additional queries for the authors to address and suggestions for text that might be helpful to rounding out the commentary if there is space for these considerations. Thank you very much for these suggestions. The final paragraph in the original manuscript was expanded to cover these issues raised by the reviewers. We also added a box with suggestions for best practices in open-source projects. We hope that the changes are adequate. We believe that the feedback that we received has greatly improved the manuscript. 1. The authors could also include that one way to overcome some of these challenges are through hands-on workshops. Some examples that seem to have been particularly successful are Optogenetic/CLARITY workshops, Miniscope workshops, DeepLabCut workshops- all demystifying open -source tools. Thank you for providing these examples and we agree that hands-on workshops are crucial to expanding open-source science (although, as mentioned in point 2 below that these are expensive and require time/effort/money/resources!). We have added some of these examples into the final section of the revised manuscript. “Hands-on workshops have been successful for several open source tools, such as optogenetics, CLARITY, Miniscope, and DeepLabCut.” 2. Another challenge that the authors may consider highlighting is that open-source development and training (such as storing data for others to download, maintaining a well-documented website and source code, as well as workshops) are expensive in both time and resources consumed to do a good job. The current reality of the limited funding opportunities to support these efforts should be noted with perhaps some guidance on possible funding sources. Thanks for this feedback. We added a statement about the need for funding to support open source efforts in the final section of the revised manuscript. “These activities will require financial support to enable storing data, designs, and protocols, maintaining a well-documented website and source code, and offering training workshops. We hope that major funders (e.g. NIH, NSF, EU) consider providing special opportunities for supporting development and training for open source research tools.”

3. Can the authors provide more insight into how common is the use of open-source tools? The number of open-source projects seems enormous, but how many of these projects actually get used? The online survey results give a kind of answer, but that's a self-selected group (people who visit openbehavior.com and are moved to answer an online survey are probably more into open source than other scientists are). One reason, I think, that people who could contribute to open source do not do so is the fear that nobody will ever use their tool, and so it'll be a waste of time. Are there any metrics on the use of open-source tools the authors could cite or describe? Unfortunately, we do not have the funding or resources to provide a large-scale study on the success of replication for open-source tools. Ideally, we would like to perform a larger analysis on this which is another reason why we would like to highlight that funding opportunities should be made available for this open-source movement. In the meantime, RRIDs were mentioned in the text and that is one way that people would be able to more easily track the use of an open-source tool, since a user could simply type in the device's RRID and find all the studies that have used that device. We expanded on the original comment in the manuscript as follows: <ORIGINAL> “... there is a need for tracking the use of open-source tools, by creating and utilizing RRIDs (SciCrunch) in publications.” <REVISED> “... there is a need for tracking the use of open-source tools, by creating and utilizing RRIDs (SciCrunch) in publications. To our knowledge, RRIDs have not been commonly created for hardware. Having a system for tracking usage has three potential impacts. First, tool usage can be tracked beyond citations of methods papers. Second, revisions and spin-offs can be noted and also tracked. Third, developers might have increased incentives to share designs early in the process, especially if an index, similar to the h factor, was developed for RRIDs.” 4. What do the authors suggest as specific approaches to establishing best practices (e.g. standards and documentation) in open-source tools? In neurophysiology, there have been efforts to establish standards for data sharing (i.e., Neurodata Without Borders). For the open source project, establishing standards (even for documentation alone) would be much more difficult, given how varied projects are, but do the authors have any ideas about how this might be done? They mention the importance of documentation/standards in the manuscript, but do they have suggestions? We would like to emphasize again the use of RRIDs as one method of best practice for open-source tools. Additionally, documentation through providing a bill of materials, a list of build instructions, and even adding in video or photo tutorials would make replication straightforward. Finally, documenting these steps on clear, user-friendly websites intended for documentation of projects such as Github, Hackaday.io, or OSF.io is ideal for a set of best practices. We would like to incorporate this in a separate box into the manuscript, if possible. This Box is noted in the final section of the revised manuscript: “See Box 1 for our recommendations for best practices in developing Open-Source tools.”

Box 1. Recommendations for best practices in developing open source tools 1. Clear documentation of the project: Provide all design files, as well as a Bill of Materials, Build Instructions, graphical (video/photo/3D renderings) descriptions or tutorials for the project. 2. Central repository for files: Provide all files and documentation of the project on a site like GitHub, Hackaday.io, OSF.io, or on the research group's website. 3. Experimental validation: Show an example of the device being used in a behavioral experiment. 4. Make the project easy to find: Create a Research Resource Identifier (RRID), using the SciCrunch project, for the device so that others can track the project across publications.

Minor comments: P. 3, I think the word should be “tracks” and not “tracts”. P.4, Teensy is normally capitalized. We have made these changes in the text and highlighted them in red.
